# Management of a giant perineal condylomata acuminata

**DOI:** 10.3205/iprs000086

**Published:** 2016-01-21

**Authors:** Evelyn Hemper, Mathias Wittau, Johannes Lemke, Marko Kornmann, Doris Henne-Bruns

**Affiliations:** 1 Clinic of General and Visceral Surgery, University of Ulm, Ulm, Germany

**Keywords:** condylomata acuminata, human papillomaviridae

## Abstract

A condylomata acuminata infection is caused by human papillomaviridae (HPV). This sexually transmitted condition most often affects the perineal region. Importantly, infections with types 16 and 18 are associated with an increased risk for anal and cervix cancer. In most cases topical therapy is sufficient for successfully treating condylomata acuminata. Here, we report the case of a 51-year old patient who suffered from a giant perianal located condylomata acuminata which had developed over a period of more than 10 years. Imaging by MRI revealed a possible infiltration of the musculus sphincter ani externus. Because a topical treatment or a radiotherapy was considered unfeasible, a surgical treatment was the only therapeutic option in this unusual case. First, a colostomy was performed and subsequently a resection of the tumor *in toto* with circular resection of the external portion of the musculus sphincter ani externus was performed. The large skin defect was closed by two gluteus flaps. The rectum wall was reinserted in the remnant of the musculus sphincter ani externus. Postoperatively, parts of the flaps developed necrosis. Therefore, a vacuum sealing therapy was initiated. Subsequently, the remaining skin defects were closed by autologous skin transplantation. Six months later the colostomy could be reversed. To date, one year after first surgery, the patient has still a normal sphincter function and no recurrence of the condylomata acuminata. This case report demonstrates how giant condylomata acuminata can be successfully treated by extended surgical procedures including colostomy and plastic reconstruction of resulting defects upon resection.

## Zusammenfassung

Condylomata acuminata werden durch humane Papillomaviren verursacht. Der Übertragungsweg erfolgt durch Schmier-und Kontaktinfektionen. Zervixkarzinome und Analkarzinome sind in 70% assoziiert mit den Subtypen der humanen Papillomaviren 16 und 18. In den meisten Fällen ist eine topische Therapie des Condylomenrasens ausreichend.

Wir berichten über einen 51-jährigen Patienten, der sich mit einem seit 10 Jahren bestehenden und progredienten perineal gelegenen Condylomenrasen vorstellte. Die Condylomata acuminata-Besiedlung war durch den Subtyp 6 des humanen Papillomavirus verursacht. Die Größe des Condylomata acuminata-Rasens betrug 16 cm in der Längsausdehnung. Im MRT des Beckens konnte eine Infiltration des Musculus ani externus nicht sicher ausgeschlossen werden. Eine topische Therapie oder eine Bestrahlung war aufgrund der Größe nicht möglich. Es erfolgte zunächst die Anlage eines doppelläufigen Descendostomas und anschließend eine Resektion des Tumors *in toto* unter Mitnahme des äußeren Anteils des Musculus ani externus. Der sehr große Hautdefekt, der beide Glutei betraf, wurde mittels zwei subkutaner Gluteusschwenklappen gedeckt. Die Mucosa des freiliegenden Musculus ani extrenus wurde zirkulär an die Haut der Gluteusschwenklappen adaptiert. Postoperativ traten am Oberrand beider Schwenklappen eine livide Verfärbung in der Breite von ca. 4 cm auf, die reseziert wurden. Diese Fläche wurde mittels einer Vakuumversiegelungstherapie behandelt. Nach 7 Wochen konnte der Defekt mittels eines Spalthaut-Meshgrafts gedeckt werden. 6 Monate später konnte das Descendostoma zurückverlagert werden. Der Patient ist kontinent und es besteht eine normale Sphinkterfunktion. Dieser Fallbericht zeigt, dass ein perinealer Condylomenrasen in dieser Größe nur durch eine chirurgische Exzision mit vorheriger Anlage eines protektiven Stomas und einer plastischen Rekonstruktion erfolgreich behandelt werden kann.

## Introduction

Condylomata acuminata is a skin manifestation caused by an infection with human papilloma viruses (HPV), in most cases by the subtypes 6, 11, 16, and 18 [1]. Condylomata acuminatum is considered as a sexually transmitted diseases since HPV infections are transmitted via skin to skin contacts [1]. Consequently, condylomata acuminata are mostly found in the perianal region and usually present as genital warts [1]. Infections with the types 6 and 11 are associated with a low risk for malignant transformation, whilst infections with subtypes 16 and 18 are associated with an increased high risk for anal cancer and cervix cancer [2]. Before initiating a therapy it is important to obtain a biopsy, to determine the risk for malignant transformation [3]. In most cases of condylomata acuminata are locally restricted and can be managed by physical ablation or by topical therapy such as aminolevulinic acid hydrochloride [4]. Only in a subset of patients with advanced condylomata acuminata more extended therapeutic strategies including radiation and even surgery is required [5]. Here, we present the case of a patients diagnosed with a giant condyloma acuminate which required complex surgical treatment with colostomy and plastic reconstruction of the resulting defects upon resection. 

## Case report

We report the case of a 51-year-old patient who had been suffering from a perianal condylomata acuminata for more than 10 years. Anamnestically, the lesions had massively increased during the last decade. 

**Figure 1:**
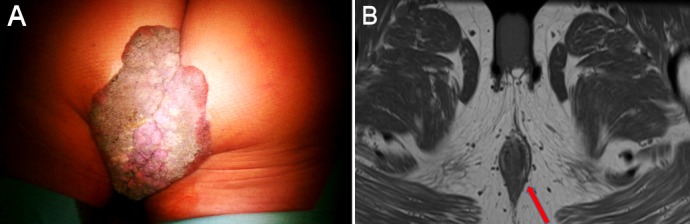
A) Preoperative aspect of the lesion (16 x 12 cm). B) Preoperative MRI: Suspect infiltration.

**Figure 2:**
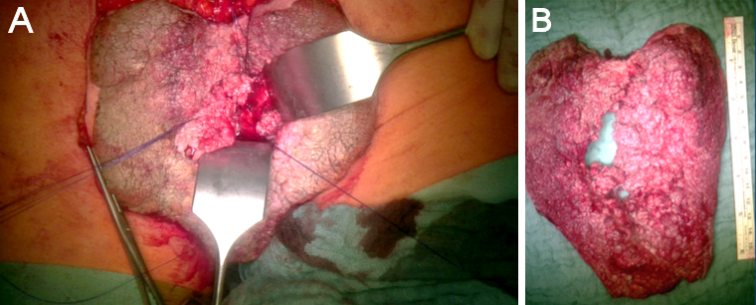
A) Situation prior the en bloc resection of the condylomata acuminate. The mucosa of the musculus sphincter ani externus is armed by sutures. B) Specimen after resection.

**Figure 3:**
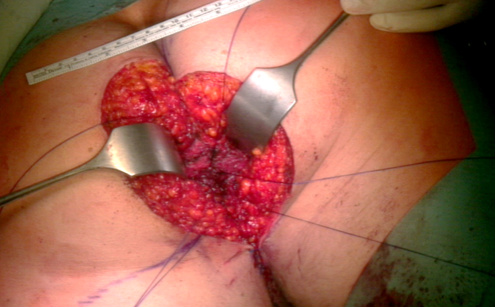
After resection, the mucosa of the musculus ani externus was fixed by circular sutures into the edge of the skin.

**Figure 4:**
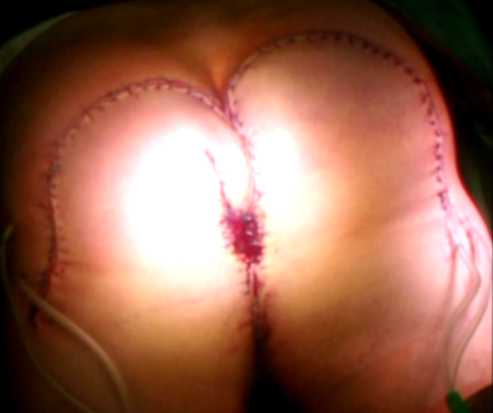
The large skin defect was closed by two gluteus flap.

**Figure 5:**
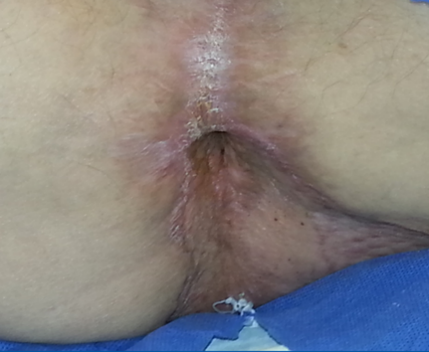
Postoperative result 6 months after the resection. The patient had a normal sphincter function. Aspect of the perianal region in prone position.

At time of presentation in our clinic the size of the condylomata acuminata amounted 16 x 12 cm (Figure 1A (Fig. 1)). The patient suffered from perianal pain and insufficient anal hygiene. An infection with HPV 6, which is associated with low risk for malignant transformation, was detected. Due to the large size of the condylomata acuminata a precise measurement of the sphincter pressure could not be performed. However, clinically the sphincter tonus appeared normally. To determine the depths of infiltration we performed a magnet resonance imaging (MRI). The MRI revealed a possible infiltration of the musculus ani externus (Figure 1B (Fig. 1)). Due to the size of the lesions a radiation did not appear beneficial in this case. Therefore a surgical treatment remained the only therapeutic option. First, a double-barreled colostomy was performed. Subsequently, the giant condylomata acuminata tumor was resected *in toto* including the resection of the external parts of the musculus sphincter ani externus (Figure 2 (Fig. 2)). The mucosa of the anus was fixed by circular sutures (Figure 3 (Fig. 3)). Because of a large lesion resulting upon resection, which measured nearly one third of the gluteus on both sides, the large defect was closed by two gluteus flaps. Finally, the mucosa of the anus was adapted to the skin of the gluteus flaps (Figure 4 (Fig. 4)). Postoperatively, the gluteus flaps became partially necrotic. Therefore repeated vacuum sealing was performed and the remaining defect was finally closed using a mesh graft. The patients was dismissed 10 weeks after surgery with completed wound healing. The patient was instructed to intensively train his musculature of the pelvic floor. Finally, 6 month later the sphincter function was sufficient and the reversal of the colostomy was performed without complications. Fortunately, the patient did not experience any defecations problems and did not develop recurrence of condylomata acuminata up to date (Figure 5 (Fig. 5)).

## Discussion

Perianal giant condylomata acuminata is a very rare clinical condition [6]. Due to improved topical therapeutic approaches, today, surgery can be circumvented in many patients [7]. However surgical therapy remains the standard therapy in cases of high risk condylomata acuminata or if topical therapy appears unfeasible due to the size and/or location of the affected lesion. Here we report the interesting case of a patient who presented with an extremely large and advanced perianal condylomata acuminata. Importantly, preoperative imaging revealed possible infiltration of the sphincter. Based on these findings, a topical therapy and/or radiation was considered unbeneficial in this case, although the condylomata acuminata was associated with a low risk HPV infection. Consequently, it is very important to perform preoperative imaging to determine infiltration depths and infiltration of other tissues in order to accurately plan the surgical strategy [7]. Interestingly, it has been demonstrated that neo-adjuvant therapy may increase the chance for achieving tumor-free resection margins in case infiltration of the sphincter [7]. However, in our case a neo-adjuvant therapy was not conducted because the infiltration of the sphincter appeared only marginal and therefore the potential benefit of a neo-adjuvant therapy appeared rather low. To support wound healing upon plastic reconstruction by two gluteus flaps we performed a colostomy prior to resection. In our case we resected the external part of the musculus ani externus and fixed the mucosa circularly into the skin of the gluteus flap. By this complex surgical procedure a “neo-anus” with full function of the sphincter was created and an abdominoperineal resection could be avoided, which had to be performed in other patients with giant condylomata acuminata [8]. Intriguingly, the patient did not develop any recurrence up to date and presented with normal sphincter function in the follow-up examination. In conclusion, we suggest an extended surgical resection of giant condylomata acuminata also with complex reconstruction of the resulting lesion as a technically feasible and in respect to the outcome promising therapeutic option for giant condylomata acuminata even with infiltration of the external sphincter. 

## Notes

### Competing interests

The authors declare that they have no competing interests.
